# Galectin-3 Enhances Vascular Endothelial Growth Factor-A Receptor 2 Activity in the Presence of Vascular Endothelial Growth Factor

**DOI:** 10.3389/fcell.2021.734346

**Published:** 2021-09-20

**Authors:** Issahy Cano, Zhengping Hu, Dina B. AbuSamra, Magali Saint-Geniez, Yin Shan Eric Ng, Pablo Argüeso, Patricia A. D’Amore

**Affiliations:** ^1^Schepens Eye Research Institute of Massachusetts Eye and Ear, Boston, MA, United States; ^2^Department of Ophthalmology, Harvard Medical School, Boston, MA, United States; ^3^Department of Pathology, Harvard Medical School, Boston, MA, United States

**Keywords:** endothelium, angiogenesis, ranibizumab, glycocalyx, migration

## Abstract

Galectin-3 (Gal3) is a carbohydrate-binding protein reported to promote angiogenesis by influencing vascular endothelial growth factor-A receptor 2 (VEGFR2) signal transduction. Here we evaluated whether the ability of Gal3 to function as an angiogenic factor involved vascular endothelial growth factor (VEGF). To address this possibility we used human retinal microvascular endothelial cells (HRECs) to determine whether exogenous Gal3 requires VEGF to activate VEGFR2 signaling and if Gal3 is required for VEGF to activate VEGFR2. VEGFR2 phosphorylation and HREC migration assays, following either VEGF neutralization with ranibizumab or Gal3 silencing, revealed that VEGF endogenously produced by the HRECs was essential for the effect of exogenous Gal3 on VEGFR2 activation and cell migration, and that VEGF-induced VEGFR2 activation was not dependent on Gal3 in HRECs. Gal3 depletion led to no reduction in VEGF-induced cell function. Since Gal3 has been suggested to be a potential therapeutic target for VEGFR2-mediated angiogenesis, it is crucial to define the possible Gal3-mediated VEGFR2 signal transduction mechanism to aid the development of efficacious therapeutic strategies.

## Introduction

Angiogenesis is the process by which blood vessels expand and remodel from existing vascular tissue. This mechanism is a critical element in embryonic development, wound healing, and disease progression (Folkman, 1995). Aberrant angiogenesis exacerbates disorders such as cancers and wet age-related macular degeneration by disturbing the well-organized vasculature and the associated vascular permeability leads to tissue dysfunction.

Vascular endothelial growth factor (VEGF), its isoforms, its receptors, and its functions have been well characterized with respect to angiogenesis. In particular, vascular endothelial growth factor-A receptor 2 (VEGFR2) mediates VEGF-induced endothelial cell (EC) survival, permeability, proliferation, tube formation, and cell migration (Koch et al., 2011; Peach et al., 2018). VEGF activation of VEGFR2 induces receptor internalization, which is an integral aspect of its signaling (Simons et al., 2016). Cell surface proteins, such as neuropilin and endomucin have been shown to mediate VEGF binding and/or VEGFR2 trafficking, influencing angiogenic signaling (Olsson et al., 2006; Park-Windhol et al., 2017; LeBlanc et al., 2019).

Galectin-3 (Gal3) is a member of a family of lectins known as galectins. Galectins have a conserved carbohydrate-recognizing domain (CRD) that has an affinity for beta-galactosides, such as those found on the extracellular domain of membrane-bound receptors, including VEGFR2 (Funasaka et al., 2014). Gal3 is considered a chimera-type galectin due to its proline-rich N-terminal domain in conjunctions with its CRD, which provides it the ability to self-oligomerize and form lattice structures at the cell surface with other plasma membrane-bound molecules (Halimi et al., 2014).

Galectin-3 has been extensively studied in cancer biology and is considered a “pro-angiogenic” molecule (Halimi et al., 2014). Gal3, along with galectin 1, have been reported to mediate VEGFR2 activity at the cell surface by enhancing its signal transduction (Markowska et al., 2011; D’Haene et al., 2013). The binding of Gal3 to VEGFR2 is independent of activation by VEGF (Markowska et al., 2011). Previous studies have reported that exogenous Gal3 induces VEGFR2 activation in ECs, following serum starvation, leading to cell migration, proliferation, and tube formation (Nangia-Makker et al., 2000; Markowska et al., 2010, 2011). The suggested mechanisms for Gal3’s activation of VEGFR2 correspond to its ability to cluster VEGFR2 at the EC surface, providing greater receptor availability to VEGF after cell starvation, as well as retention of the receptor at the cell surface. Most current anti-angiogenic treatments are focused on VEGF. However, due to challenges such as side effects seen with their use in cancer treatment and non-responder and/or resistance observed with anti-VEGF therapies in the eye, alternative and/or complementary therapies are being sought. Gal3’s involvement in angiogenesis has led to the suggestion that it may provide an alternative anti-angiogenic target. This study aims to elucidate further the functional relationship between VEGF and Gal3 in VEGFR2 activation.

## Materials and Methods

### Reagents and Antibodies

#### Reagents

VEGF165 was purchased from Cell Signaling (#8065SC). Ranibizumab was obtained from Genentech. Mouse IgG (#sc-2025) was purchased from Santa Cruz. Goat (#005-000-003) and rabbit (#111-005-003) IgG was obtained from Jackson ImmunoResearch Laboratories. Phosphatase inhibitor cocktail tablet (#4906845001), protease inhibitor cocktail table (#5892970001), primaquine bisphosphate (PQB, #160393-1G), and Tween-20 (#X251-07) were purchased from Sigma-Aldrich. Sulfo-NHS-SS-Biotin (#1859385) and avidin agarose (#S1258122) were purchased from Life Technologies. Cell Lysis Buffer (#9803S) was from Cell Signaling. Gal3 was produced using vector as described previously (Mauris et al., 2013). Phosphate buffered saline (PBS, Sigma-Aldrich #D5652-10 × 1 L) and Tris-buffered saline (TBS, #170-6435) were obtained from Bio-Rad. Sodium orthovanadate was acquired from Sigma-Aldrich (#S6508-10G).

#### Antibodies

Immunoblots were probed with rabbit anti-Gal3 (1:1,000, Abcam #ab209344), rabbit anti-VEGFR2 (1:1,000, Cell Signaling, #2479S) mouse anti-CD31 (1:1,000, Thermo Fisher, #14-0311-81), rabbit anti-phospho-Y1175-VEGFR2 (1:500, Cell Signaling, #2478S), mouse anti-α-tubulin (1:1,000, Millipore, #CP06-100UG), and goat anti-VEGFR2 (for immunocytochemistry, 1:100, R&D System, #AF357). Secondary antibodies used for immunoblots include goat anti-rabbit 800CW (1:20,000, LI-COR, #925-32211) and goat anti-mouse 680RD (1:20,000, LI-COR, #925-68070).

### Cell Culture

Human retinal microvascular endothelial cells (HRECs) were purchased from Cell Systems (#ACBRI 181) and cultured in Endothelial Basal Media-2 (EBM-2) BulletKit Medium (Lonza, #CC-3162) supplemented with 2% fetal bovine serum (FBS, Atlanta Biologicals) and 2 mM L-glutamine (Lonza, #CC-17-605E). Culture plates were coated in 0.2% gelatin from porcine skin (Sigma-Aldrich, #G1890) for 30 min at 37°C. Cells were used up to passage nine.

#### siRNA Knockdown

Human retinal microvascular endothelial cells were seeded at 70–80% confluence 8 h before siRNA application. siControl (siCtrl) (50 nM, Ambion #4390844) or siGal3 (50 nM, Ambion #4392422) was incubated for 30 min at room temperature with Lipofectamine (Thermo Fisher, #L3000001) in OPTIMEM (Life Technologies, #51985034). The siRNA complex was added to the HRECs with 2% FBS complete EBM-2 media without penicillin–streptomycin overnight and removed with a change in medium.

### Cell Migration

Confluent HRECs were starved for 8 h in serum-free EBM-2 then mechanically scratched with a p200 pipette tip. Serum-free EBM-2 containing the Gal3 (50 ng/ml) with mouse IgG or ranibizumab (10 μg/ml) was applied to the cells. Images were taken right after scratching and 15 h after to obtain an area of the closure. Wound area was analyzed using the Image J MRI Wound Healing Tool plug-in.

### Biotin Cell Surface Isolation

Confluent HRECs were starved for 2 h in serum free EBM-2, cells were stimulated for 30 min with BSA (10 ng/ml), VEGF (10 ng/ml), or Gal3 (50 ng/ml) in the presence of mouse IgG or ranibizumab (10 μg/ml) in serum free EBM-2 with primaquine bisphosphate (0.6 μM). Sulfo-NHS-SS-Biotin (#1859385) in PBS was added for 30 min at 4°C. The cells were washed with 50 mM Tris (pH 8.0), followed by PBS (pH 8.0) for quenching. Cells were collected using TBS and centrifuged at 1,500 rcf for 10 min. The cell pellet was lysed and sonicated in cell lysis buffer then incubated with 100 μl of avidin agarose bead slurry (#S1258122), rotating for 1 h at room temperature. The beads were then washed using a Wash Buffer (20 mM Tris–HCl, pH 6.8, 0.5% Tween-20) containing a protease inhibitor cocktail (#5871S) three times, centrifuged at 1,000 rcf for 1 min. Bound cell surface proteins were eluted using Laemmli SDS Sample Buffer with 100 mM DTT followed by a 10 min incubation at 95°C. Western blot analysis was performed on the samples with antibodies against rabbit anti-human VEGFR2 (1:1,000; #AF357) and mouse anti-human CD31 (1:1,000; #14-0311-81). The Dual Color Precision Plus Protein^TM^ from Bio-Rad was used to identify the molecular weights of proteins (Supplementary Figure 1).

### Measurement of Kinase Activation

Human retinal microvascular endothelial cells grown to confluence were starved in serum-free EBM-2 for 2 h then stimulated with BSA (10 ng/ml), Gal3 (50 μg/ml), or VEGF (10 ng/ml) in serum-free EBM-2 with 200 mM sodium orthovanadate for 0, 5, 10, 30, and 60 min. After washing the cells in PBS, they were collected in lysis buffer and analyzed by western blot for tubulin, VEGFR2, and pVEGFR2.

### Analysis of Protein Expression

Following lysis in RIPA buffer (Cell Signaling, #9806S) with protease and phosphatase inhibitor cocktails (1:100, Sigma), samples were prepared with equal protein concentration determined using Peirce BCA assay kit (Thermo Scientific, #23227), run on SDS-PAGE, transferred onto nitrocellulose membranes (VWR, #27376-991), washed with 0.1–0.2% Tween-20 in PBS (PBST). Membranes were probed with antibodies for the proteins of interest. Corresponding secondary antibodies were used. SuperSignal^TM^ West Pico Chemiluminescent Substrate (Thermo Scientific, #34077) or fluorescence LI-COR Odyssey (LI-COR) was utilized for final image development. Densitometric measurements were determined using ImageJ software. Final protein fluorescence was normalized to loading controls and represented as a fold-change.

### qPCR RNA Analysis

Immediately after treatment, cells were washed with PBS, collected and processed using the RNeasy Mini Kit provided by Qiagen. A measured amount of 1,000 ng of purified RNA was reverse transcribed into cDNA using the iScript cDNA synthesis kit (Bio-Rad). Using FastStart Universal SYBR Green master mix (Roche #A25743), gene expression was read with the Precision Plus Protein^TM^ LightCycler^®^ 480 II (Roche, Indianapolis, IN, United States) on 384 well plates containing a starting amount of 25 ng of cDNA. The primers used included:

E-selectin: Forward (5′-GCTGGAGAACTTGCGTTTAAG-3′)
Reverse (5′-GCTTTGCAGACTGGGATTTG-3′)

V-CAM1: Forward (5′-CCACGTGGACATCTACTCTTTC-3′)
Reverse (5′-CCAGCCTGTAAACTGGGTAAA-3′)

HPRT: Forward (5′-CCTGGCGTCGTGATTAGTGAT-3′)
Reverse (5′-AGACGTTCAGTCCTGTCCATAA-3′)

PrimePCR^TM^ PCR primers for Gal3 were acquired from Bio-Rad (#qHsaCID0008552). Gene expression levels were normalized to HPRT. All samples were prepared with the same amount of starting reagents for qPCR analysis.

### Statistical Analysis

Data are presented as the mean ± SEM of at least three independent experiments. To evaluate for statistical significance, two-tail unpaired Student *T*-test or original one-way ANOVA were used (Prism 9 software package, GraphPad, San Diego, CA, United States). Values of *P* < 0.05 were considered statistically significant.

## Results

### Galectin-3 Promotes Cell Function Through Vascular Endothelial Growth Factor-A Receptor 2

**FIGURE 1:**
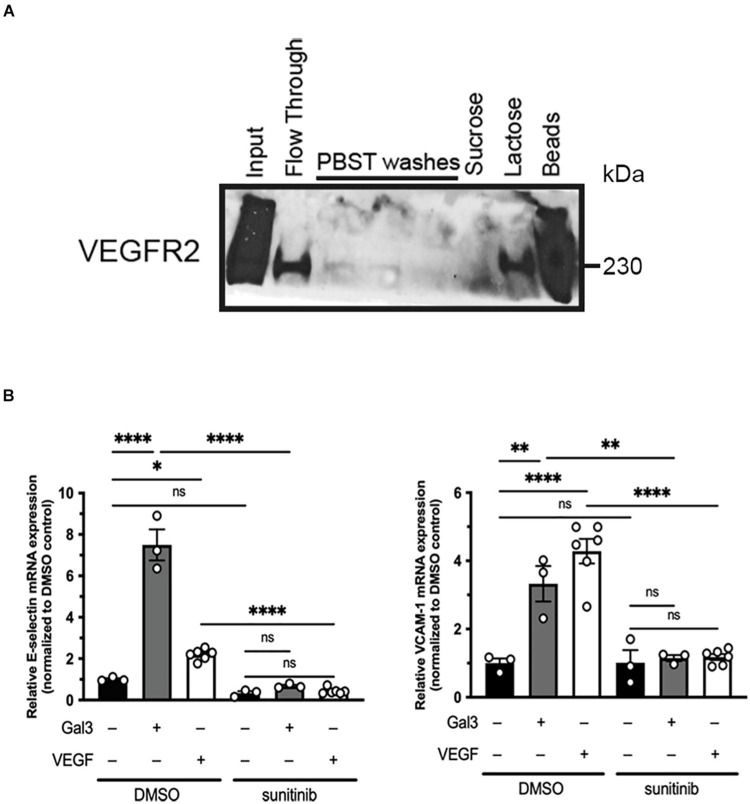
Galectin-3 associates with and induces activation of VEGFR2. **(A)** Confluent HRECs were lysed using RIPA lysis buffer. HREC lysate was incubated with Gal3-conjugated beads, washed, and eluted either with a non-competitive sugar (0.1 mM sucrose) or a competitive sugar (0.5 mM lactose). Bound VEGFR2 was eluted once it was competed off of the beads by lactose **(B)** Serum-starved HRECS were stimulated with 50 μg/ml Gal3 or 10 ng/ml VEGF for 2 h with or without sunitinib then total RNA was isolated and reverse transcribed into cDNA. E-selectin, VCAM-1, and HPRT1 mRNA expression were analyzed using qPCR. One-way ANOVA analysis was applied, ^∗^*P* < 0.05, ^∗∗^*P* < 0.01, ^****^P < 0.0001, *n* = 3.

Galectin-3 has been reported to interact with VEGFR2 *via N*-glycans on its extracellular domain (Markowska et al., 2011). We confirmed this by examining Gal3 and VEGFR2 association using lysates of HREC and Gal3-conjugated agarose beads (Figure 1A). Bound materials eluted with lactose and analyzed by western blot revealed the binding of VEGFR2 by Gal3. These data suggest that Gal3 does in fact bind to VEGFR2 in a galactose-dependent manner.

In addition to its effects as an angiogenesis factor, VEGFR2 also mediates the pro-inflammatory signaling of VEGF (Kim et al., 2001; Shaik-Dasthagirisaheb et al., 2013). Thus, as an additional endpoint, we examined the expression of the leukocyte adhesion molecules, E-selectin and VCAM-1 (Chen et al., 2013). Gal3 stimulation of serum starved HRECs led to an increase in E-selectin (7.46 ± 0.745 vs. 1.0 ± 0.051, *P* < 0.001) and VCAM-1 (3.329 ± 0.519 vs. 1.0 ± 0.140, *P* < 0.05) mRNA, compared to untreated controls (Figure 1B). Sunitinib, a small molecule inhibitor that is selective for VEGFR2, was used to confirm the role of VEGFR2 in this effect. Serum starved HRECs treated with sunitinib had significantly reduced Gal3-induction of E-selectin by 91% (0.676 ± 0.0601 vs. 7.46 ± 0.745, *P* < 0.001) and VCAM-1 by 67% (1.158 ± 0.0908 vs. 3.329 ± 0.519, *P* < 0.05) mRNA expression (Figure 1B), providing strong evidence for the involvement of VEGFR2 in the effects of Gal3. EC migration, a component of angiogenesis, is induced by VEGF *via* activation of VEGFR2 (Santos et al., 2007).

### Galectin-3 Enhances, but Is Not Required for Vascular Endothelial Growth Factor-Induced Cell Function

**FIGURE 2:**
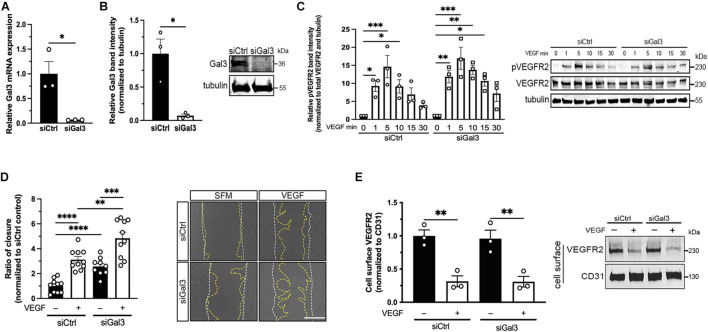
Galectin-3 is not required for VEGF-induced VEGFR2 activity. **(A)** HRECs transfected with siRNA targeting Gal3 (siGal3) demonstrated over 90% mRNA knockdown of Gal3 compared to cells transfected with control siRNA (siCtrl), analyzed by reverse-transcription and qPCR assay. Student *T*-test was applied, ^∗^*P* < 0.05, *n* = 3. **(B)** Lysate from HRECs transfected with siGal3 or siCtrl was analyzed by western blot and probed with an antibody against Gal3, which resulted in over 90% Gal3 protein reduction. Student *T*-test was applied, ^∗^*P* < 0.05, *n* = 3. **(C)** siCtrl and siGal3 transfected HRECs were stimulated with 10 ng/ml of VEGF for 0, 1, 5, 10, 15, and 30 min. Cell lysates were collected and analyzed by western blot for tubulin, VEGFR2, and phospho-VEGFR2 (pVEGFR2 Y1175). Protein levels for pVEGFR2 were quantified using ImageJ and normalized to total VEGFR2 and α-tubulin. Ordinary one-way ANOVA was applied for comparisons within groups, ^∗^*P* < 0.05, ^∗∗^*P* < 0.01, ^∗∗∗^*P* < 0.001, *n* = 3. **(D)** Confluent HRECs, transfected with siCtrl or siGal3, were scratched and stimulated with 10 ng/ml of VEGF. Images were taken immediately after scratching and 15 h later. Cell migration was quantified using ImageJ and normalized to the non-treatment control group. Scale bar represents 500 μm. Student *T*-test was applied, ^∗∗^*P* < 0.01, ^∗∗∗^*P* < 0.001, ^****^P < 0.0001, *n* = 10. **(E)** HRECs transfected with siCtrl and siGal3 were stimulated with 10 ng/ml VEGF. Cell surface proteins were labeled with NHS-SS-biotin and isolated from cell lysate using avidin agarose beads. Total VEGFR2 and CD31 protein levels were analyzed by western blot. Student *T*-test was applied, ^∗^*P* < 0.05, *n* = 3.

After establishing the association between Gal3 and VEGFR2, we sought to determine if Gal3 was necessary for VEGF-induced VEGFR2 activity. Treatment of HRECs with siGal3 resulted in more than 90% reduction of Gal3 mRNA (0.0643 ± 0.00231 vs. 1.0 ± 0.247, *P* < 0.05) (Figure 2A) and protein compared to the siCtrl group (0.072 ± 0.0233 vs. 1.0 ± 0.218, *P* < 0.05) (Figure 2B). The depletion of Gal3 on the endothelial cell surface was also observed using immunocytochemistry (Supplementary Figure 2). Stimulation of HRECs in which Gal3 had been knocked down with 10 ng/ml VEGF over a time course up to 30 min revealed no significant difference in the phosphorylation of VEGFR2. The phosphorylation of VEGFR2 at all time points was similar in both siCtrl and siGal3 conditions (Figure 2C). Consistent with that observation, Gal3 depletion did not prevent VEGF-stimulated HREC migration; VEGF-induced migration in Gal3 depleted cells was significantly compared to control cells (4.85 ± 0.474 vs. 2.31 ± 0.352 *P* < 0.01) (Figure 2D).

Vascular endothelial growth factor activation of VEGFR2 leads to receptor internalization through clathrin-medicated endocytosis (LeBlanc et al., 2019). To assess whether Gal3 is necessary for this step, HRECs with depleted Gal3 were stimulated with VEGF at 10 ng/ml, all cell surface proteins were biotinylated and extracted from whole cell lysate with avidin agarose beads, then were analyzed for total VEGFR2 levels on the cell surface by western blot. There was no difference in VEGF-induced VEGFR2 internalization with or without Gal3 depletion (0.0143 ± 0.00373 vs. 0.0146 ± 0.00381, *P* > 0.05) (Figure 2E).

### Vascular Endothelial Growth Factor Is Necessary for Gal3-Induced Vascular Endothelial Growth Factor-A Receptor 2 Activation

**FIGURE 3:**
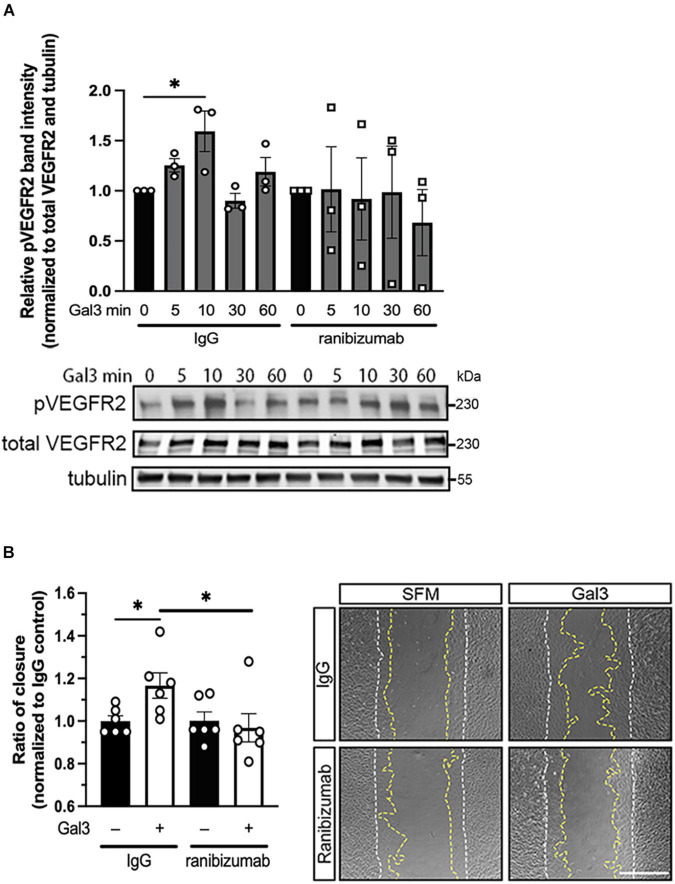
Galectin-3 requires VEGF to induce VEGFR2 activity. **(A)** Ranibizumab (10 μg/ml) was added to neutralize endogenous VEGF and HRECs were stimulated with 50 μg/ml of Gal3 for 0, 5, 10, 30, and 60 min. Cell lysates were collected and pVEGFR2 Y1175 (pR2), VEGFR2 (TR2), and α-tubulin protein levels were analyzed by western blot. Ordinary one-way ANOVA was applied. ^∗^*P* < 0.05, *n* = 3. **(B)** Confluent HRECs were scratched and stimulated with 50 μg/ml of Gal3 with 10 μg/ml of ranibizumab or control IgG. Images were taken immediately after scratching and at 15 h later. Scale bar represents 500 μm. Cell migration was quantified using ImageJ. Student *T*-test was applied, ^∗^*P* < 0.05, *n* = 6.

In light of our observation that Gal3 appears to enhance the activities of VEGFR2, we sought to determine the role of VEGF in Gal3’s induction of VEGFR2 signaling. To accomplish this, we employed ranibizumab, a recombinant humanized antibody fragment (Fab) that neutralizes all VEGF-A isoforms. Serum-starved HRECs were stimulated with Gal3 in the presence of ranibizumab to neutralize VEGF in the system, and VEGFR2 phosphorylation (pVEGFR2 Y115) was examined over a time course. Results showed that ranibizumab blocked the action of Gal3 as an inducer of VEGFR2 activation (Figure 3A). Gal3 induced significant VEGFR2 phosphorylation without ranibizumab in the control cells at the early time point (1.59 ± 0.202 vs. 1.0, *P* < 0.05). Treatment with ranibizumab to neutralize VEGF completely inhibited Gal3-induced VEGFR2 phosphorylation, and ranibizumab alone had no effect on VEGFR2 activation (0.764 ± 0.249 vs. 1.0 ± 0.236, *P* > 0.05).

To determine if VEGF is essential for the downstream effects of Gal3, we examined HREC migration. The scratch migration assay was conducted using Gal3 along with ranibizumab or mouse IgG as a control. HRECs treated with Gal3 in the presence of a control IgG migrated significantly more than the untreated control cells by about 17% (1.167 ± 0.0235 vs. 1.0 ± 0.310, *P* < 0.05). VEGF neutralization by ranibizumab completely blocked the stimulatory effect of Gal3 on HREC migration (0.968 ± 0.207 vs. 1.0 ± 0.140, *P* > 0.05) (Figure 3B).

Taken together these results reveal that Gal3 does not directly active VEGFR2 but depends on the presence of VEGF endogenously produced by the HREC.

## Discussion

Galectin-3 has been previously shown to play an influential role in angiogenesis, in particularly in the context of VEGFR2 signaling. Surprisingly, Gal3 induced the activation of VEGFR2 even after the ECs were starved in serum-free media. As previously suggested, this phenomenon may be due to Gal3’s ability to enhance VEGFR2 signaling in the presence of the suboptimal VEGF ligand rather than directly activating VEGFR2 itself (Markowska et al., 2011). The results of this study support the conclusion that while VEGF is necessary for the activation of VEGFR2-mediated signaling and functions, Gal3 facilitates that interaction but does not directly signal through VEGFR2.

Galectin-3 plays diverse roles in the physiology and function of cells, so not surprisingly, is implicated in a variety of pathologies. Elevated serum levels of Gal3 have been detected during the progression of various head and neck carcinomas, as well as in acute inflammation and renal failure (Nishiyama et al., 2000; Saussez et al., 2008; Henderson and Sethi, 2009). In contrast, downregulation of Gal3 in prostate cancer was shown to favor cancer progression (Pacis et al., 2000) and decreased expression of Gal3 in the context of breast cancer is speculated to contribute to metastasis (Castronovo et al., 1996). In addition, Gal3 knockdown has been previously shown to reduce VEGF- and basic fibroblast growth factor (FGF)-mediated angiogenesis (Markowska et al., 2010). In this work we determined that Gal3 depletion did not lead to a reduction in VEGF-mediated VEGFR2 phosphorylation at the Y1175 site, VEGF-induced HREC migration, or VEGF-induced VEGFR2 internalization. Thus, that while Gal3 is not necessary for VEGF-induced VEGFR2 activity, it does appear to modulate it by enhancing it as it has been previously reported (Markowska et al., 2010). VEGFR2 is not the only tyrosine kinase receptor that Gal3 binds. FGF receptor 1 and epidermal growth factor receptor are N-glycosylated at the extracellular domain and, similar to VEGFR2, bind to Gal3 in a carbohydrate dependent manner (Duchesne et al., 2006; Azimzadeh Irani et al., 2017; Kucinska et al., 2019). In doing so, Gal3 has the potential to enhance their angiogenic signaling as well, potentially using the same or similar mechanism. FGF modulation by Gal3 was studied alongside VEGF and both were impacted similarly (Markowska et al., 2010).

Previous reports have examined the role of Gal3 in VEGFR2 signal transduction. Addition of Gal3 to human umbilical vein ECs resulted in the phosphorylation of VEGFR2 at the Y1175 (Markowska et al., 2011), which could lead to the conclusion that Gal3 functions as a ligand to directly activate VEGFR2 and downstream signal transduction. However, as suggested, any presence of VEGF, even post serum-starvation, may be amplified by the presence of Gal3, possibly *via* its effect on receptor clustering (Markowska et al., 2011). Our observation that neutralization of VEGF prevented the ability of exogenously added Gal3 to induce VEGFR2 signal transduction support this notion and that Gal3 acts to amplify angiogenic signaling in an indirect manner.

Anti-VEGF therapies have transformed treatment strategies for pathologies involving aberrant angiogenesis. Ranibizumab along with bevacizumab and aflibercept are potent anti-VEGF treatments aimed at mitigating disease progression, such as in neovascular age-related macular degeneration. However, a significant number of patients show a variable response to conventional anti-VEGF therapy, and some are considered non-responders (Kitchens et al., 2013; Krebs et al., 2013; Bontzos et al., 2020). The findings in this study that Gal3 acts as an enhancer to VEGF activity suggest that blocking both VEGF and Gal3 might lead to a more robust inhibitory effect.

## Data Availability Statement

The original contributions presented in the study are included in the article/Supplementary Material, further inquiries can be directed to the corresponding author.

## Author Contributions

IC performed the research, analyzed the data, and wrote the first draft of the manuscript. ZH, DA, MS-G, YN, and PA provided critical discussion and technical help for the experiments and reviewed the manuscript. IC, ZH, MS-G, YN, and PD’A designed the research. PD’A directed the study, interpreted results with IC, and did the major editing of the manuscript. All authors contributed to the article and approved the submitted version.

## Conflict of Interest

The authors declare that the research was conducted in the absence of any commercial or financial relationships that could be construed as a potential conflict of interest.

## Publisher’s Note

All claims expressed in this article are solely those of the authors and do not necessarily represent those of their affiliated organizations, or those of the publisher, the editors and the reviewers. Any product that may be evaluated in this article, or claim that may be made by its manufacturer, is not guaranteed or endorsed by the publisher.

## Supplementary Material

Supplementary Figure 1 Western blot with protein standard present. A representative western blot was chosen to demonstrate the positioning of the protein standard. The standard used is the Dual Color Precision Plus Protein^TM^ Standards from Bio-Rad, which shows a maximum kDa of 250 and a minimum kDa of 10.

Supplementary Figure 2 Immunofluorescence of cell surface Gal3. HRECs were grown to confluency on gelatin-coated coverslips. Prior to VEGF (10 ng/ml) stimulation, HRECs were starved in serum-free EBM-2 for 2 h. The HRECs were washed with PBS and fixed with 1% paraformaldehyde overnight at 4°C. The non-permeabilized cells were stained for DAPI, Gal3, and VEGFR2 followed by incubation with the appropriate fluorescent secondary antibodies [Alexa Fluor 594-labeled donkey anti-goat (1:1,000, #A-11058) and Alex Fluor 488-labeled donkey anti-rabbit (1:1,000, #A-21206)]. Images at 20× magnification were taken using the Zeiss Axioscope (Oberkochen, Germany). The scale bar represents 100 μm. Expression of Gal3 and VEGFR2 was confirmed on the cell surface, as well as the knockdown of Gal3 using siRNA.
