# Supplementary figures and images for: Galectin-3 Enhances Vascular Endothelial Growth Factor-A Receptor 2 Activity in the Presence of Vascular Endothelial Growth Factor
Source: Front Cell Dev Biol. 2021 Sep 20;9:734346. doi: 10.3389/fcell.2021.734346 (PMC8488270; doi:10.3389/fcell.2021.734346)

Supplemental Figure 1.

CD31

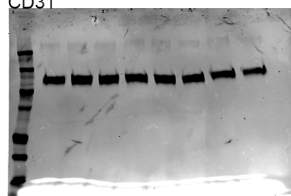

VEGFR2

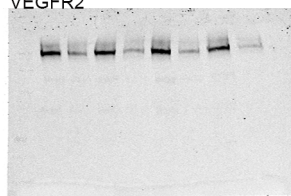

Supplement: Supplementary Figure 1 — Western blot with protein standard present. A representative western blot was chosen to demonstrate the positioning of the protein standard. The standard used is the Dual Color Precision Plus ProteinTM Standards from Bio-Rad, which shows a maximum kDa of 250 and a minimum kDa of 10. [file Data_Sheet_1.PDF]

Supplemental Figure 2.

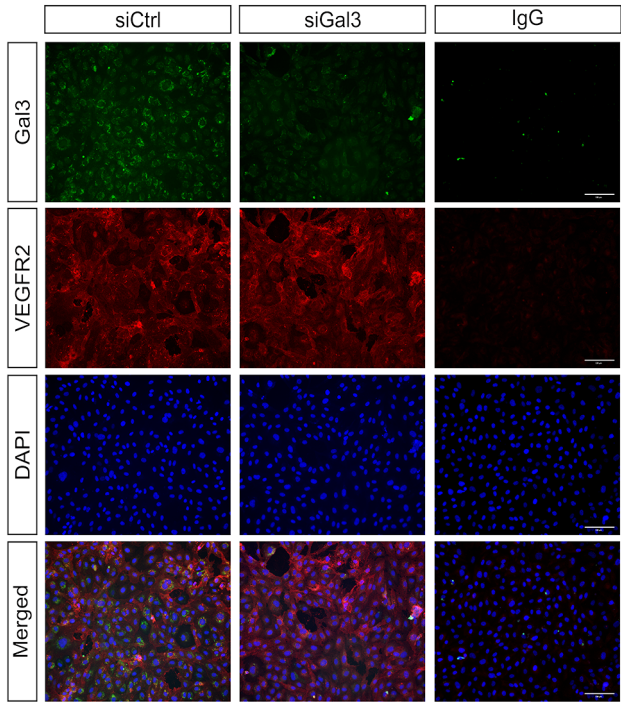

Supplement: Supplementary Figure 2 — Immunofluorescence of cell surface Gal3. HRECs were grown to confluency on gelatin-coated coverslips. Prior to VEGF (10 ng/ml) stimulation, HRECs were starved in serum-free EBM-2 for 2 h. The HRECs were washed with PBS and fixed with 1% paraformaldehyde overnight at 4°C. The non-permeabilized cells were stained for DAPI, Gal3, and VEGFR2 followed by incubation with the appropriate fluorescent secondary antibodies [Alexa Fluor 594-labeled donkey anti-goat (1:1,000, #A-11058) and Alex Fluor 488-labeled donkey anti-rabbit (1:1,000, #A-21206)]. Images at 20× magnification were taken using the Zeiss Axioscope (Oberkochen, Germany). The scale bar represents 100 μm. Expression of Gal3 and VEGFR2 was confirmed on the cell surface, as well as the knockdown of Gal3 using siRNA. [file Data_Sheet_2.PDF]
